# Sex Differences in Reverse Left Ventricular Remodeling in Patients Who Underwent Transcatheter Aortic Valve Replacement in a Chinese Population

**DOI:** 10.31083/RCM39581

**Published:** 2025-08-28

**Authors:** Jiaqi Zhang, Chengwei Chi, Li Cha, Yuwei Wang, Yuxin Shao, Qingtao Meng, Shulong Zhang, Jihong Liu, Enze Jin

**Affiliations:** ^1^Department of Cardiology, Harbin Medical University, 150000 Harbin, Heilongjiang, China; ^2^Cardiovascular Medical Department, The Fourth Affiliated Hospital of Harbin Medical University, 150000 Harbin, Heilongjiang, China; ^3^Department of Cardiology, Affiliated Zhongshan Hospital of Dalian University, 116000 Dalian, Liaoning, China

**Keywords:** aortic stenosis, transcatheter aortic valve replacement, sex differences, left ventricular remodeling, echocardiography

## Abstract

**Background:**

Differences between female and male patients may influence the outcomes of transcatheter aortic valve replacement (TAVR). However, knowledge regarding known sex differences in TAVR procedures among Chinese people remains limited. Therefore, this study aimed to investigate the impact of sex-related differences on reverse left ventricular (LV) remodeling following TAVR in the Chinese population.

**Methods:**

Patients with severe symptomatic aortic stenosis (AS) who underwent TAVR at the Heart Center of the Affiliated Zhongshan Hospital of Dalian University were enrolled. A total of 136 patients who underwent implantation of a self-expandable Venus A valve between 2019 and 2024 were evaluated. We retrospectively compared the clinical outcomes and characteristics of all patients by sex.

**Results:**

In our study, females presented with a smaller body surface area (BSA) (1.68 ± 0.15 m^2^ vs. 1.90 ± 0.14 m^2^, *p* < 0.001), aortic valve area (AVA) (0.64 ± 0.22 cm^2^ vs. 0.77 ± 0.20 cm^2^, *p* = 0.003), left ventricular end-diastole diameter (LVEDD) (49.72 ± 7.37 mm vs. 53.33 ± 8.36 mm, *p* = 0.023), as well as interventricular septum in diastole (IVSD) (12.85 ± 2.19 mm vs. 13.88 ± 2.61 mm, *p* = 0.034) at baseline. Comparatively, males had larger aortic root structures at baseline and a larger size of valve implantation during the procedure (*p* < 0.05). However, the indexed AVA was not significantly different between the two groups at baseline. Sex-specific outcomes, particularly AVA, LVEDD, aortic root diameter (AO), and IVSD, were significantly different during each follow-up within the first six months (*p* < 0.05), indicating that females experienced greater improvements in these echocardiographic characteristics after TAVR. Left ventricular ejection fraction (LVEF) only improved significantly at 1-month follow-up in females compared to males (57.77 ± 7.87% vs. 54.40 ± 8.21%, *p* = 0.037). Multivariable linear-regression analysis showed that being a female patient (Beta: 10.200; 95% CI: 0.075–20.326; *p* = 0.048), as well as having a higher IVSD (Beta: 2.939; 95% CI: 1.110–4.769; *p* = 0.002), and higher baseline left ventricular mass index (LVMi) (Beta: 0.409; 95% CI: 0.298–0.521; *p* < 0.001) were independently associated with greater mid-term LVMi regression post-TAVR.

**Conclusions:**

Female patients with AS exhibited more favorable mid-term LV reverse remodeling post-TAVR compared to male patients in a Chinese population.

## 1. Introduction

Transcatheter aortic valve replacement (TAVR) is considered to be an established percutaneous replacement for severe aortic stenosis (AS) in patients at high risk or inoperable patients [1, 2, 3, 4, 5]. More recently, it has been suggested that TAVR is noninferior to surgical aortic valve replacement (SAVR) in patients at low risk and intermediate risk [2, 3, 4]. Recent data suggest that sex-based differences in clinical outcomes do exist. Better midterm and long-term survival after TAVR are associated with female patients despite higher periprocedural complication rates, particularly increased vascular complications. In major TAVR studies, females make up roughly half of the study population. Understanding the outcome characteristics in the female population is crucial, as females often exhibit different tolerance to severe AS compared to males, leading to distinct ventricular remodeling patterns [6, 7, 8]. Left ventricular (LV) reverse remodeling, which is related to mid-term and long-term prognosis, is highly important [9, 10, 11].

Although recent publications have explored sex-specific factors associated with TAVR, the clinical outcomes have not been fully elucidated. A brief study of 305 consecutive TAVR patients revealed no sex-related differences in mortality at 30 days but did detect higher risk of bleeding or periprocedural vascular complication rates in females [12, 13]. AS induces left ventricular overload, resulting in adverse remodeling characterized by cardiac muscle hypertrophy and interstitial collagen deposition, ultimately impairing diastolic and systolic ventricular function. These changes play an important role in LV remodeling [14, 15]. This clinical outcome was associated with sex-related differences, with females exhibiting less myocardial fibrosis, more concentrated LV geometry and better myocardial systolic function [16]. Another observational study of 92 severe AS patients who underwent TAVR, which included 53 females, demonstrated that females experienced faster regression. That study concluded that females could adapt to pressure overload better and could recover faster than males [17]. Further testing of gene and biopsy data from patients with severe LV septal hypertrophy revealed that males had more LV fibrosis than females did [16]. An increase in profibrotic genes may explain why males have reverse less LV remodeling after TAVR.

Several studies have examined sex-related differences in TAVR procedures in Western populations [18, 19, 20, 21]. However, little is known about TAVR outcomes stratified by sex in the Chinese population. Thus, the aim of this study was to evaluate whether there are any sex-related differences in outcomes or reverse LV remodeling in a Chinese population.

## 2. Material and Methods

### 2.1 Study Population

A total of 136 patients with severe AS who underwent TAVR at the Heart Center of Affiliated Zhongshan Hospital of Dalian University between 2019 and 2024 were retrospectively evaluated. All patients who underwent TAVR were selected and reviewed by a dedicated team comprised of experienced cardiac surgeons and interventional cardiologists. Assessments of medical history, transesophageal or transthoracic echocardiography, and thoracic computed tomography were used to evaluate AS. These patients were deemed either inoperable or at high risk for SAVR after discussion with the dedicated team. The follow-up data of each patient were collected during a clinical visit or a standardized phone call at one month and six months post-discharge after TAVR. The study was approved by the local ethics committee of the Affiliated Zhongshan Hospital of Dalian University. No annexed industry funding was supplied. This study was driven by the interests of the investigators. Written informed consent was obtained from all patients prior to TAVR, and the study conformed to the Declaration of Helsinki.

### 2.2 TAVR Procedure

TAVR was performed with a self-expanding prosthesis, the Venus A valve (Venus MedTech, Inc., Hangzhou, China). The self-expanding prosthesis possesses a refined supra-annular design devoid of an outer skirt. Retightening, repositioning and even retrieval were not allowed in the delivery catheter system. The Venus A valve has been also widely used in the Chinese population because of the design of a trileaflet valve, which is a made of porcine pericardial tissue and lacks an outer skirt. A 20-F sheath delivery system (Version 20-F Braidin™ Pro guiding catheter; APT Medical, Xiangtan, China) was used to accommodate the prosthesis. A strong radial force was the most dominant feature of the prosthesis and was more suitable for the Chinese population’s calcified anatomy [22]. Three valve sizes were widely used: 23, 26, and 29 mm. The transfemoral approach was the preferred access, besides method, in addition to the transapical approach and transaxillary approach. Several patients were unsuitable for the procedure through the iliofemoral artery. In most cases, percutaneous coronary intervention (PCI) was conducted before the TAVR procedures. PCI was conducted in the same procedural session as TAVR in only 2 male and 1 female patients. The key inclusion criteria were as follows: (1) severe AS patients diagnosed on the recommendation of the European Society of Cardiology/European Association for Cardio-Thoracic Surgery Guidelines (aortic valve area ≤1.0 cm^2^/aortic valve index ≤0.6 cm^2^/m^2^/peak aortic velocity (Vmax) ≥4.0 m/s); (2) patients at intermediate- to high-risk surgical risk or a Society of Thoracic Surgeons risk (STS) score >4; and (3) patients with severe AS with typical symptoms. The main exclusion criteria were as follows: (1) patients with active endocarditis, acute aortic dissection, or acute myocardial infarction; (2) patients with expectations of life <1 year.

### 2.3 Echocardiography

Transthoracic echocardiographic follow-up was performed at baseline (pre-TAVR), at hospital discharge, and at one and 6 months after TAVR. Two-dimensional Doppler transthoracic echocardiography was expertly performed with a Phillips EPIQ 7 system (Phillips, Eindhoven, Netherlands) by the same echocardiologist. Standard parasternal long-axis views, short-axis views, 4-chamber views, and 2-chamber views were obtained. The Bernoulli simplified equation was used for calculating the mean pressure gradient (MPG) using continuous wave Doppler. The left ventricular end-diastole diameter (LVEDD), interventricular septum in diastole (IVSD), and left ventricular posterior wall thickness (LVPWT) were measured in two dimensions with the parasternal view on the basis of guideline recommendations [23, 24]. The LV mass index (LVMi) was calculated based on Deveraux’s formula in accordance with the joint recommendations of the European Association of Echocardiography companied with and the American Society of Echocardiography as follows: relative wall thickness (RWT) = (LVPWT × 2)/LVEDD. LV mass (LVM) = 0.8 × 1.04 × [(LVEDD + IVSD + LVPWT)^3^ – LVEDD^3^] + 0.6. LVMi = LVM/body surface area (BSA) [25]. The definition of left ventricle hypertrophy (LVH) was an LVMi exceeding 125 g/m^2^ in men and 110 g/m^2^ in women [26].

### 2.4 Statistical Analysis

All continuous data are expressed as the means ± standard deviations (SD) or medians as well as interquartile ranges, as appropriate, while categorical variables are presented as numbers and percentages. Categorical variables were compared using the Chi-squared or Fisher’s exact tests, as appropriate. Paired (for before and after comparisons) and unpaired (for independent group comparisons) Student’s *t* tests were used for normally distributed data. The Mann‒Whitney or Wilcoxon signed-rank test was used for nonparametric data. SPSS Version 25.0 (IBM Corp., Armonk, NY, USA) was used for data analysis, and a *p* value < 0.05 was considered to indicate statistical significance.

To evaluate the changes of repeated echocardiographic characteristics between males and females, a global and mixed-effects model (main effect of sex and time, and interaction) has now been assessed using repeated-measures two-way ANOVAs, depending on the variable. The primary hypotheses focused on the difference at baseline, 24 hours after TAVR, 1-month follow-up and 6-month follow-up between the male group and female group. In a secondary analysis, the follow-up time point was also included. The follow-up was 24 hours, 1-month, and 6-month after TAVR procedures. Group allocation (male vs. female) was the between-group factor. Time was the within-group factor (24 hours, 1-month, 6-month). Compared with the same group at baseline, ^a^*p* < 0.05, Compared with the same group at 24 hours after TAVR, ^b^*p* < 0.05, Compared with the same group at 1 M follow-up after TAVR, ^c^*p* < 0.05, Compared with the same group at 6 M follow-up after TAVR, ^d^*p* < 0.05, *p* values in bold are statistically significant. If the sphericity assumption was violated, the conservative Greenhouse–Geisser correction was applied. Global effects of sex and time were tested, and post hoc pairwise comparisons were conducted using Bonferroni correction to adjust for multiple comparisons.

Multivariable linear regression models were constructed to identify independent factors associated with left ventricular remodeling parameters. Adjustment variables were selected based on a two-step approach: (1) clinical relevance supported by previous literature; and (2) statistical significance in univariate analysis with a threshold of *p* < 0.15. Variables meeting either criterion were considered for inclusion in the final model. The F-test is commonly used to evaluate whether a linear regression model is statistically significant overall. A significant F-test (*p* < 0.05) indicates that the model explains a meaningful proportion of the variance in the dependent variable, and at least one predictor has a statistically significant relationship with the outcome.

## 3. Results

### 3.1 Baseline and Procedural Data of Patients

**Fig. 1.**
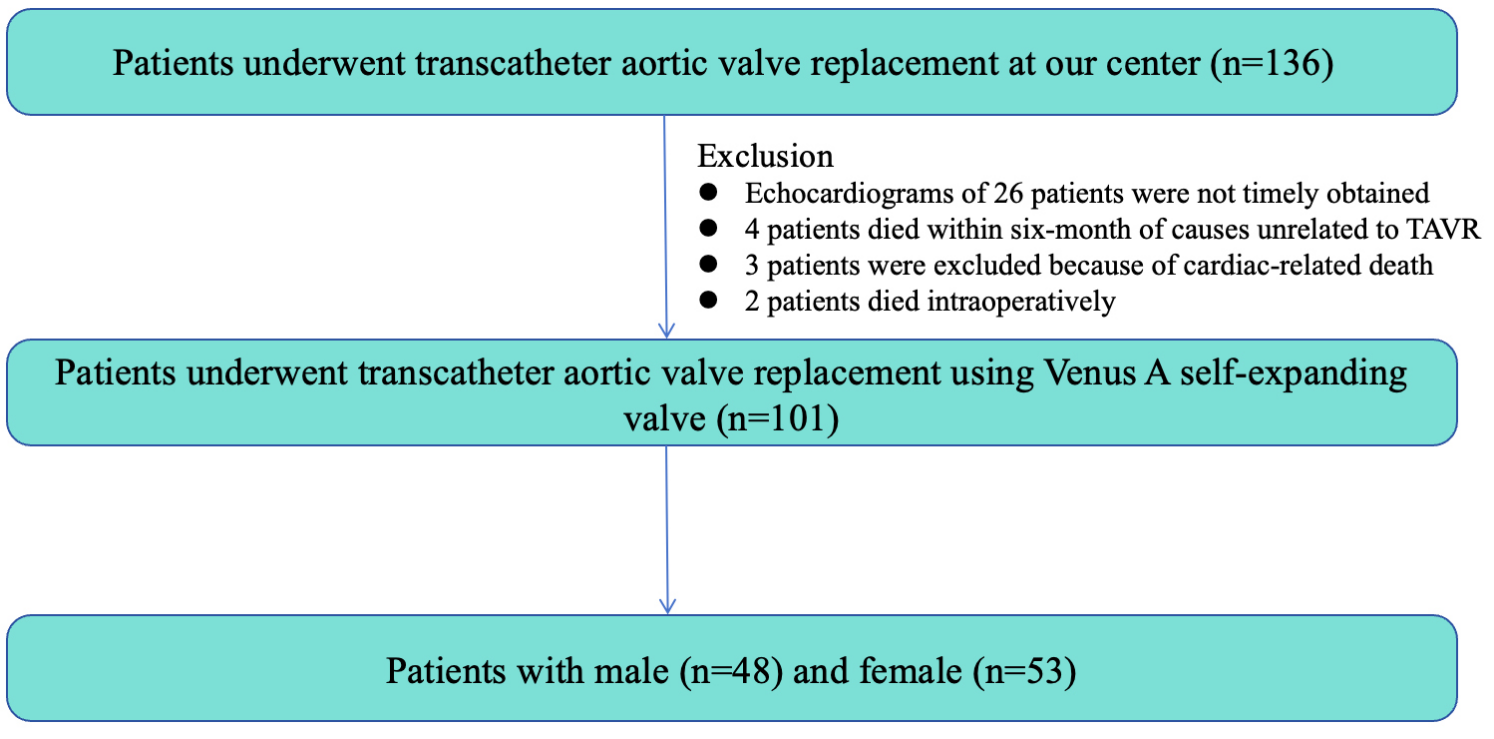
**Study flow chart**. TAVR, transcatheter aortic valve replacement.

As shown in Fig. 1, of the 136 patients who were referred for enrollment in our study, 35 were excluded. Echocardiograms of 26 patients were not obtained in a timely manner after TAVR during the six-month follow-up because of follow-up at a referral hospital or poor image quality. Four patients died within six months of a cause unrelated to the TAVR procedure. Three patients were excluded because of cardiac-related death during the six-month follow-up. Two patients died during the procedure. The remaining 101 patients (n = 48 men and 53 women) fulfilled the study criteria.

The baseline characteristics are listed in Table 1. The mean age was 74.5 ± 8.3 years, and 52.5% were females. The BSA was 1.78 ± 0.18 m^2^, and females were significantly smaller (1.68 ± 0.15 m^2^ vs. 1.90 ± 0.14 m^2^, *p* < 0.001) than males. Females had significantly less dyslipidemia (47.2% vs. 72.9%, *p* = 0.009) and less peripheral vascular disease (PVD) (20.8% vs. 39.6%, *p* = 0.039) than males did. No significant differences in the electrocardiogram at baseline were observed between the two male and female patients. Higher rate of coronary artery disease (CAD) (58%) was found in our study. As shown in Table 1, no significant differences of prevalence of CAD were observed between the male and female patients ([30 (62.5) vs. 28 (52.8)], *p* = 0.326) at baseline. Comorbidities, including diabetes, hypertension, chronic obstructive pulmonary disease (COPD), stroke, atrial fibrillation, previous valvular replacement surgery, and previous coronary artery bypass grafting (CABG), were comparable for both male and female patients.

**Table 1.** **Baseline characteristics**.

	All (n = 101)	Male (n = 48)	Female (n = 53)	*p* value
Age (years, $\bar{x}$ ± *s*)	74.5 ± 8.3	74.3 ± 8.8	74.7 ± 7.9	0.771
BSA (m^2^, $\bar{x}$ ± *s*)	1.78 ± 0.18	1.90 ± 0.14	1.68 ± 0.15	< **0.001**
NYHA Class III/IV	81 (80.1)	40 (83.3)	41 (77.4)	0.452
CrCl (mL/minute, $\bar{x}$ ± *s*)	79.55 ± 35.42	83.02 ± 37.09	76.40 ± 33.89	0.351
Comorbidities:				
Dyslipidemia	60 (59.4)	35 (72.9)	25 (47.2)	**0.009**
Diabetes	37 (36.6)	18 (37.5)	19 (35.8)	0.863
Hypertention	63 (62.4)	30 (62.5)	33 (62.3)	0.981
COPD	5 (5.0)	5 (10.4)	0	0.051
Stroke	43 (42.6)	17 (35.4)	26 (49.1)	0.166
PVD	30 (29.7)	19 (39.6)	11 (20.8)	**0.039**
CAD	58 (57.4)	30 (62.5)	28 (52.8)	0.326
Atrial fibrillation	31 (30.7)	16 (33.3)	15 (28.3)	0.584
Previous valvular replacement surgery	3 (3.0)	2 (4.2)	1 (1.9)	0.931
Prior CABG	2 (2.0)	1 (2.1)	1 (1.9)	1.000
Need for urgent aortic valvular intervention	1 (1.0)	1 (2.1)	1 (1.9)	1.000
Risk evaluation:				
EuroScore II ($\bar{x}$ ± *s*)	6.89 ± 6.95	6.55 ± 6.24	7.08 ± 7.59	0.708
STS mortality ($\bar{x}$ ± *s*)	3.97 ± 3.37	3.73 ± 3.75	4.18 ± 3.00	0.511
STS morbimortality ($\bar{x}$ ± *s*)	14.54 ± 7.57	13.21 ± 7.24	15.74 ± 7.74	0.094
Electrocardiogram:				
Sinus	79 (78.2)	37 (77.1)	42 (79.2)	0.800
Atrial fibrillation	17 (16.8)	10 (20.8)	7 (13.2)	0.303
Other atrial rhythm	4 (4.0)	2 (4.2)	2 (3.8)	1.000
Abnormal cardiac electric axis	54 (53.5)	25 (52.1)	29 (54.7)	0.803
1° AVB	16 (15.8)	10 (20.8)	6 (11.3)	0.203
LBBB	4 (4.0)	2 (4.2)	2 (3.8)	1.000
RBBB	12 (11.9)	7 (14.6)	5 (9.4)	0.443
LAFB	5 (5.0)	5 (10.4)	0	0.053
Echocardiogram:				
MPG (mmHg, $\bar{x}$ ± *s*)	50.19 ± 24.21	47.35 ± 18.46	52.75 ± 28.38	0.265
AVA (cm^2^, $\bar{x}$ ± *s*)	0.70 ± 0.22	0.77 ± 0.20	0.64 ± 0.22	**0.003**
AVA/BSA (cm^2^/m^2^, $\bar{x}$ ± *s*)	0.39 ± 0.12	0.41 ± 0.11	0.38 ± 0.13	0.308
LVEF (%, $\bar{x}$ ± *s*)	52.23 ± 12.42	51.52 ± 12.20	52.87 ± 12.70	0.589
LVEDD (mm, $\bar{x}$ ± *s*)	51.44 ± 8.02	53.33 ± 8.36	49.72 ± 7.37	**0.023**
AO (mm, $\bar{x}$ ± *s*)	20.44 ± 2.55	21.33 ± 2.79	19.62 ± 2.00	**0.001**
LVPWT (mm, $\bar{x}$ ± *s*)	12.12 ± 2.13	12.46 ± 2.26	11.81 ± 1.97	0.128
IVSD (mm, $\bar{x}$ ± *s*)	13.34 ± 2.44	13.88 ± 2.61	12.85 ± 2.19	**0.034**
LVMi (g/m^2^, $\bar{x}$ ± *s*)	151.17 ± 41.37	157.36 ± 42.75	145.56 ± 39.65	0.153
RWT (cm, $\bar{x}$ ± *s*)	0.51 ± 0.13	0.51 ± 0.14	0.51 ± 0.12	1.000

BSA, body surface area; NYHA, New York Heart Association; CrCl, creatinine clearance; COPD, chronic obstructive pulmonary disease; PVD, peripheral vascular disease; CAD, coronary artery disease; CABG, coronary artery bypass graft; STS, Society of Thoracic Surgeons; 1° AVB, first-degree atrioventricular block; LBBB, left bundle branch block; RBBB, right bundle branch block; LAFB, left anterior fascicular block; MPG, mean pressure gradient; AVA, aortic valve area; LVEF, left ventricular ejection fraction; LVEDD, left ventricular end-diastolic dimension; AO, aortic root diameter; LVPWT, left ventricular posterior wall thickness; IVSD, interventricular septum in diastole; LVMi, left ventricular mass index; RWT, relative wall thickness; *p* values in bold are statistically significant.

Table 1 shows the evaluation of the echocardiographic parameters before the TAVR procedure. At baseline, no significant differences were found between the two groups based on the MPG. Additionally, the aortic valve area indexed to the body surface area (AVA/BSA), left ventricular ejection fraction (LVEF), LVPWT, and RWT were comparable between males and females. This study did not find any marked sex-related differences in the LVMi, with the exception of the AVA, LVEDD, IVSD or aortic root diameter (AO). More females had a smaller AVA (0.64 ± 0.22 cm^2^ vs. 0.77 ± 0.20 cm^2^, *p* = 0.003), a smaller LVEDD (49.72 ± 7.37 mm vs. 53.33 ± 8.36 mm, *p* = 0.023) and a smaller AO (19.62 ± 2.00 mm vs. 21.33 ± 2.79 mm, *p* = 0.001) than males did.

Procedural data of two groups were analyzed in Table 2. As shown in Table 2, no significant differences in paravalvular leak were observed between the two male and female patients. During the procedure, more females were more likely to undergo implantation of size of Venus-A valve and a smaller aortic annulus diameter (22.97 ± 3.43 mm vs. 24.97 ± 2.46 mm, *p* = 0.001) than males did, as shown in Table 2.

**Table 2.** **Procedural data**.

		All (n = 101)	Male (n = 48)	Female (n = 53)	*p* value
Venus-A valve size					**0.037**
	23 mm	24 (23.8)	11 (22.9)	13 (24.5)	
	26 mm	53 (52.5)	20 (41.7)	33 (62.3)	
	29 mm	22 (21.8)	15 (31.3)	7 (13.2)	
	32 mm	2 (2.0)	2 (4.2)	0	
Aortic annulus diameter (mm, $\bar{x}$ ± *s*)		23.91 ± 3.16	24.97 ± 2.46	22.97 ± 3.43	**0.001**
Mild paravalvular leak		7 (6.9)	2 (4.2)	5 (9.4)	0.517
Moderate/severe paravalvular leak		7 (6.9)	4 (8.3)	3 (5.7)	0.892

*p* values in bold are statistically significant.

### 3.2 Doppler Echocardiographic Data at Discharge, at 1-Month and 6-Month Follow-Up Between Males and Females

**Fig. 2.**
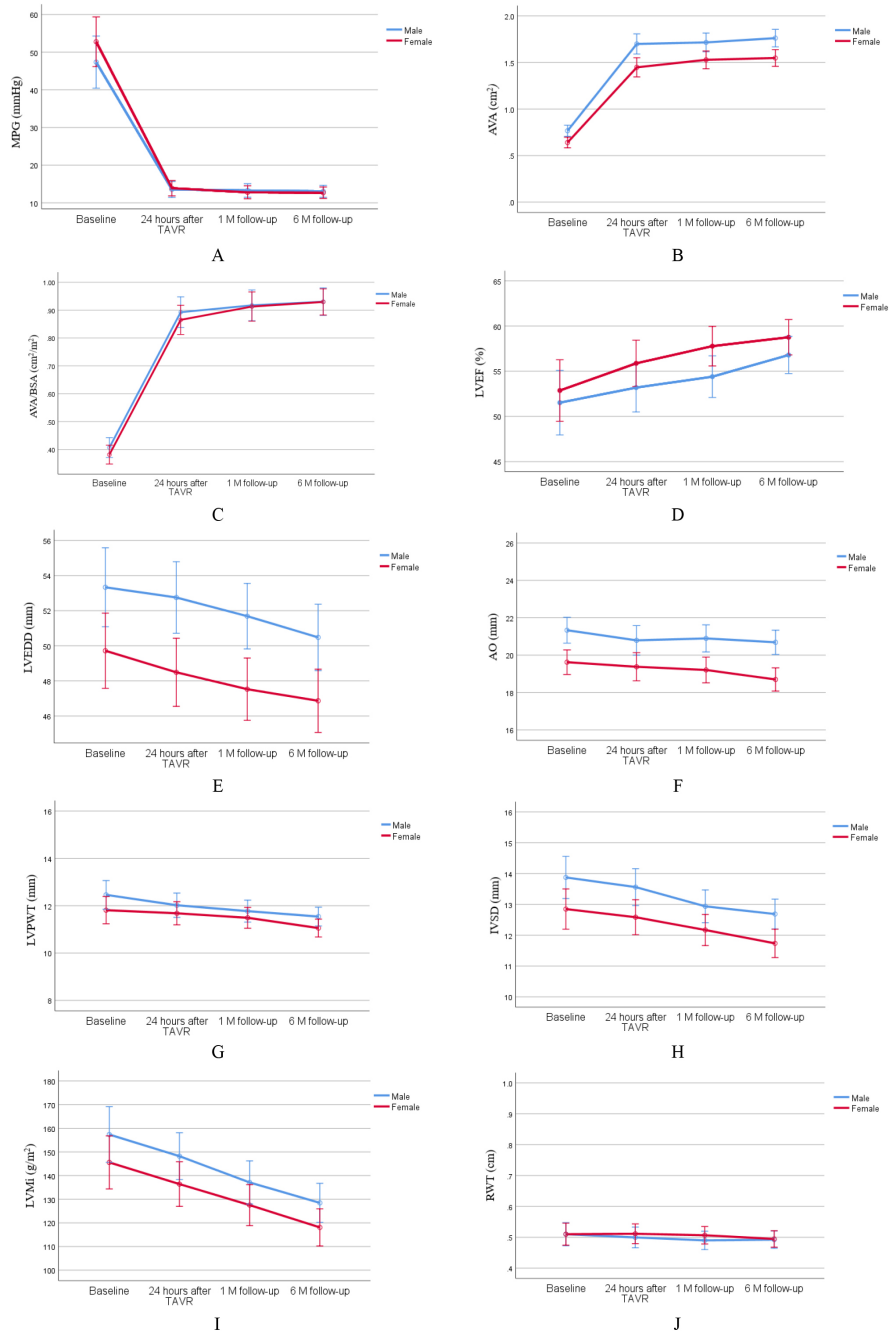
**Changes of echocardiographic characteristics at six-month follow-up post-TAVR in male and female patients**. Changes in mean pressure gradient (A), aortic valve area (B), aortic valve area/body surface area (C), left ventricular ejection fraction (D), left ventricular end-diastolic dimension (E), aortic root diameter (F), left ventricular posterior wall thickness (G), interventricular septum in diastole (H), left ventricle mass index (I) and relative wall thickness (J) at six-month follow-up post-TAVR in male and female patients. MPG, mean pressure gradient; AVA, aortic valve area; BSA, body surface area; LVEF, left ventricular ejection fraction; LVEDD, left ventricular end-diastolic dimension; AO, aortic root diameter; LVPWT, left ventricular posterior wall thickness; IVSD, interventricular septum in diastole; LVMi, left ventricular mass index; RWT, relative wall thickness; TAVR, transcatheter aortic valve replacement.

As shown in Table 3, the patterns of echocardiographic characteristics before and after TAVR were analyzed. The AVA in females was significantly smaller than that in males during every postoperative follow-up [24 hours after TAVR: (1.45 ± 0.35) cm^2^ vs. (1.70 ± 0.41) cm^2^, *p* = 0.001; one-month after TAVR: (1.53 ± 0.35) cm^2^ vs. (1.72 ± 0.35) cm^2^, *p* = 0.008; six-month after TAVR: (1.55 ± 0.35) cm^2^ vs. (1.76 ± 0.31) cm^2^, *p* = 0.002]. Even if AVA pre-and post-procedure is bigger in males, there was no significant differences between the two groups in regard to indexed values, since males also have higher BSA values than females. The main changes of echocardiographic characteristics in males and females are shown in Fig. 2 during the postoperative follow-up.

**Table 3.** **Echocardiographic characteristics: before the procedure, at discharge, at one-month follow-up and at six-month follow-up**.

		Male (n = 48)	Female (n = 53)	F value	*p* value
MPG (mmHg, $\bar{x}$ ± *s*)					
	Baseline	47.35 ± 18.46	52.75 ± 28.38	1.256	0.265
	24 hours after TAVR	13.56 ± 7.25^a^	13.89 ± 7.61^a^	0.048	0.827
	1 M follow-up	13.29 ± 6.69^a^	12.81 ± 5.89^a^	0.147	0.702
	6 M follow-up	13.06 ± 5.80^a^	12.70 ± 5.32^a^	0.108	0.743
F value		38.396	57.984		
*p* value		< **0.001**	< **0.001**		
Global test					
	sex (F value, *p* value)	0.468, 0.496			
	time (F value, *p* value)	263.221, <**0.001**			
	sex*time (F value, *p* value)	1.531, 0.207			
AVA (cm^2^, $\bar{x}$ ± *s*)					
	Baseline	0.77 ± 0.20	0.64 ± 0.22	9.299	**0.003**
	24 hours after TAVR	1.70 ± 0.41^a^	1.45 ± 0.35^a^	1.585	**0.001**
	1 M follow-up	1.72 ± 0.35^a^	1.53 ± 0.35^a^	0.882	**0.008**
	6 M follow-up	1.76 ± 0.31^a^	1.55 ± 0.35^a^	1.148	**0.002**
F value		122.245	113.104		
*p* value		< **0.001**	< **0.001**		
Global test					
	sex (F value, *p* value)	14.884, <**0.001**			
	time (F value, *p* value)	395.596, <**0.001**			
	sex*time (F value, *p* value)	1.296, 0.276			
AVA/BSA (cm^2^/m^2^, $\bar{x}$ ± *s*)					
	Baseline	0.41 ± 0.11	0.38 ± 0.13	1.052	0.308
	24 hours after TAVR	0.89 ± 0.19^a^	0.87 ± 0.20^a^	0.510	0.477
	1 M follow-up	0.92 ± 0.19^a^	0.91 ± 0.20^a^	0.013	0.908
	6 M follow-up	0.93 ± 0.15^a^	0.93 ± 0.19^a⁢b^	0.001	0.977
F value		113.061	134.324		
*p* value		< **0.001**	< **0.001**		
Global test					
	sex (F value, *p* value)	0.317, 0.575			
	time (F value, *p* value)	385.871, <**0.001**			
	sex*time (F value, *p* value)	0.275, 0.843			
LVEF (%, $\bar{x}$ ± *s*)					
	Baseline	51.52 ± 12.20	52.87 ± 12.70	0.294	0.589
	24 hours after TAVR	53.19 ± 8.99	55.87 ± 9.74^a^	2.053	0.155
	1 M follow-up	54.40 ± 8.21	57.77 ± 7.87^a⁢b^	4.455	**0.037**
	6 M follow-up	56.79 ± 7.16^a⁢b⁢c^	58.75 ± 7.17^a⁢b^	1.891	0.172
F value		6.560	6.999		
*p* value		< **0.001**	< **0.001**		
Global test					
	sex (F value, *p* value)	1.912, 0.170			
	time (F value, *p* value)	24.180, <**0.001**			
	sex*time (F value, *p* value)	0.831, 0.478			
LVEDD (mm, $\bar{x}$ ± *s*)					
	Baseline	53.33 ± 8.36	49.72 ± 7.37	5.340	**0.023**
	24 hours after TAVR	52.75 ± 7.99	48.49 ± 6.22^a^	9.030	**0.003**
	1 M follow-up	51.69 ± 7.50^a^	47.53 ± 5.46^a^	10.277	**0.002**
	6 M follow-up	50.48 ± 7.68^a⁢b⁢c^	46.87 ± 5.45^a⁢b^	7.531	**0.007**
F value		4.731	5.447		
*p* value		**0.004**	**0.002**		
Global test					
	sex (F value, *p* value)	8.769, **0.004**			
	time (F value, *p* value)	20.490, <**0.001**			
	sex*time (F value, *p* value)	0.402, 0.751			
AO (mm, $\bar{x}$ ± *s*)					
	Baseline	21.33 ± 2.79	19.62 ± 2.00	12.689	**0.001**
	24 hours after TAVR	20.79 ± 2.75	19.38 ± 2.77	6.612	**0.012**
	1 M follow-up	20.90 ± 3.03	19.21 ± 1.96	11.261	**0.001**
	6 M follow-up	20.69 ± 2.78	18.70 ± 1.69^a⁢c^	19.295	< **0.001**
F value		1.586	57.984		
*p* value		0.198	**0.003**		
Global test					
	sex (F value, *p* value)	16.484, <**0.001**			
	time (F value, *p* value)	4.270, **0.006**			
	sex*time (F value, *p* value)	0.572, 0.634			
LVPWT (mm, $\bar{x}$ ± *s*)					
	Baseline	12.46 ± 2.26	11.81 ± 1.97	2.362	0.128
	24 hours after TAVR	12.02 ± 2.00^a^	11.68 ± 1.59	0.913	0.342
	1 M follow-up	11.77 ± 1.68^a^	11.49 ± 1.56	0.754	0.280
	6 M follow-up	11.54 ± 1.53^a⁢b^	11.06 ± 1.25^a⁢b⁢c^	3.076	0.485
F value		6.236	6.125		
*p* value		**0.001**	**0.001**		
Global test					
	sex (F value, *p* value)	1.890, 0.172			
	time (F value, *p* value)	18.968, <**0.001**			
	sex*time (F value, *p* value)	1.016, 0.386			
IVSD (mm, $\bar{x}$ ± *s*)					
	Baseline	13.88 ± 2.61	12.85 ± 2.19	4.621	**0.034**
	24 hours after TAVR	13.56 ± 2.18	12.58 ± 2.00	5.533	**0.021**
	1 M follow-up	12.94 ± 1.91^a⁢b^	12.17 ± 1.81^a⁢b^	4.319	**0.040**
	6 M follow-up	12.69 ± 1.86^a⁢b^	11.74 ± 1.52^a⁢b⁢c^	7.991	**0.006**
F value		8.497	8.053		
*p* value		< **0.001**	< **0.001**		
Global test					
	sex (F value, *p* value)	6.322, **0.014**			
	time (F value, *p* value)	31.205, <**0.001**			
	sex*time (F value, *p* value)	0.374, 0.772			
LVMi (g/m^2^, $\bar{x}$ ± *s*)					
	Baseline	157.36 ± 42.75	145.56 ± 39.65	2.069	0.153
	24 hours after TAVR	148.20 ± 36.97^a^	136.40 ± 32.27^a^	2.933	0.090
	1 M follow-up	137.10 ± 35.25^a⁢b^	127.51 ± 28.43^a⁢b^	2.280	0.134
	6 M follow-up	128.46 ± 33.52^a⁢b⁢c^	118.09 ± 23.79^a⁢b⁢c^	3.260	0.074
F value		18.796	17.800		
*p* value		< **0.001**	< **0.001**		
Global test					
	sex (F value, *p* value)	2.896, 0.092			
	time (F value, *p* value)	73.769, <**0.001**			
	sex*time (F value, *p* value)	0.149, 0.930			
RWT (cm, $\bar{x}$ ± *s*)					
	Baseline	0.51 ± 0.14	0.51 ± 0.12	0.000	1.000
	24 hours after TAVR	0.50 ± 0.12	0.51 ± 0.11	0.262	0.610
	1 M follow-up	0.49 ± 0.11	0.51 ± 0.10	0.641	0.425
	6 M follow-up	0.49 ± 0.10	0.49 ± 0.09	0.014	0.905
F value		1.340	1.087		
*p* value		0.266	0.358		
Global test					
	sex (F value, *p* value)	0.134, 0.715			
	time (F value, *p* value)	2.472, 0.089			
	sex*time (F value, *p* value)	0.707, 0.490			

MPG, mean pressure gradient; AVA, aortic valve area; BSA, body surface area; LVEF, left ventricular ejection fraction; LVEDD, left ventricular end-diastolic dimension; AO, aortic root diameter; LVPWT, left ventricular posterior wall thickness; IVSD, interventricular septum in diastole; LVMi, left ventricular mass index; RWT, relative wall thickness; TAVR, transcatheter aortic valve replacement. Column 2 and 3: compared with the same group at baseline, ^a^*p* < 0.05, compared with the same group at 24 hours after TAVR, ^b^*p* < 0.05, compared with the same group at 1 M follow-up after TAVR, ^c^*p* < 0.05, *p* values in bold are statistically significant; Column 5: compared with the male and female groups at the same follow-up time, *p* < 0.05, *p* values in bold are statistically significant; Down (*p* value): compared with the same group in total (at baseline, 24 hours after TAVR, 1 M follow-up or 6 M follow-up), *p* values in bold are statistically significant in the same group; Down (global test: sex: *p* value): compared with the male and female groups in total (at baseline, 24 hours after TAVR, 1 M follow-up or 6 M follow-up), *p* < 0.05; Down (global test: time: *p* value): compared with each follow-up time, *p* < 0.05; Down (global test: sex*time: *p* value): compared with each follow-up time, *p* > 0.05; not significant in the interaction between sex and time; If the sphericity assumption was violated, the conservative Greenhouse–Geisser correction was applied; adjustments for multiple pairwise comparisons were applied using the Bonferroni correction.

### 3.3 Doppler Echocardiographic Data of Patients at Discharge, at 1-Month and 6-Month Follow-Up Compared With Pre-TAVR Values

For both males and females, as shown in Table 3, the improvements in the MPG, AVA, AVA/BSA and LVMi post-TAVR were significant at every postoperative follow-up compared to those pre-TAVR at baseline (^a^*p* < 0.05). No significant difference was found in the interaction between sex and time.

For male patients, the improvements in the LVPWT post-TAVR were significant at every postoperative follow-up compared to those pre-TAVR at baseline (^a^*p* < 0.05). LVEDD and IVSD at 24 hours after TAVR presented no significant differences in males compared to pre-TAVR values (*p* > 0.05), but they improved significantly at the 1-month and 6-month follow-up compared to pre-TAVR values (^a^*p* < 0.05). At 24 hours post-TAVR, the 1-month follow-up, LVEF in males remained similar with the values observed before TAVR. Significant difference in LVEF was found at the 6-month follow-up post-TAVR in males (^a^*p* < 0.05), as shown in Table 3.

In the male group, the remodeling in the IVSD and LVMi were significant at 1-month post-TAVR compared with baseline (^a^*p* < 0.05) and 24 hours follow-up (^b^*p* < 0.05). The reduction of LVEF, LVEDD and LVMi were significantly in male patients at 6-month follow-up compared with follow-up from baseline to 1-month post-TAVR (^a^*p* < 0.05 compared with baseline; ^b^*p* < 0.05 compared with 24 hours follow-up; ^c^*p* < 0.05 compared with 1-month follow-up). The improvement of LVPWT and IVSD were significantly in male patients at 6-month follow-up compared with baseline (^a^*p* < 0.05) and 24 hours follow-up (^b^*p* < 0.05), as shown in Table 3.

Furthermore, in the Female group, the remodeling in the LVEF, LVEDD were significant at every postoperative follow-up compared to those pre-TAVR at baseline (^a^*p* < 0.05). IVSD improved significantly at 1-month and 6-month follow-up compared to pre-TAVR values (^a^*p* < 0.05). Both AO and LVPWT during 24 hours after TAVR and the 1-month follow-up showed no significant differences in females, but they improved significantly at the 6-month follow-up compared to pre-TAVR values (^a^*p* < 0.05), as shown in Table 3.

For female patients, the remodeling in the LVEF, IVSD and LVMi were significant at 1-month post-TAVR compared with baseline (^a^*p* < 0.05) and 24 hours follow-up (^b^*p* < 0.05). The reduction of LVPWT, IVSD and LVMi were significantly in female patients at 6-month follow-up compared with follow-up from baseline to 1-month post-TAVR (^a^*p* < 0.05 compared with baseline; ^b^*p* < 0.05 compared with 24 hours follow-up; ^c^*p* < 0.05 compared with 1-month follow-up). The reduction of AVA/BSA, LVEF and LVEDD were significantly in female patients at 6-month follow-up compared with follow-up from baseline to 24 hours post-TAVR (^a^*p* < 0.05 compared with baseline; ^b^*p* < 0.05 compared with 24 hours follow-up). The improvement of AO was significantly in female patients at 6-month follow-up compared with baseline (^a^*p* < 0.05 compared with baseline) and 1-month follow-up (^c^*p* < 0.05).

Sex-specific outcomes, especially AVA, LVEDD, AO, IVSD were significantly different during each follow-up within six-month (*p* < 0.05), which indicated that females had greater improvements after TAVR in those echocardiographic characteristics. LVEF only improved significantly at 1-month follow-up in females compared to males (57.77 ± 7.87% vs. 54.40 ± 8.21%, *p* = 0.037), as shown in Table 3.

### 3.4 LV Reverse Remodeling

**Fig. 3.**
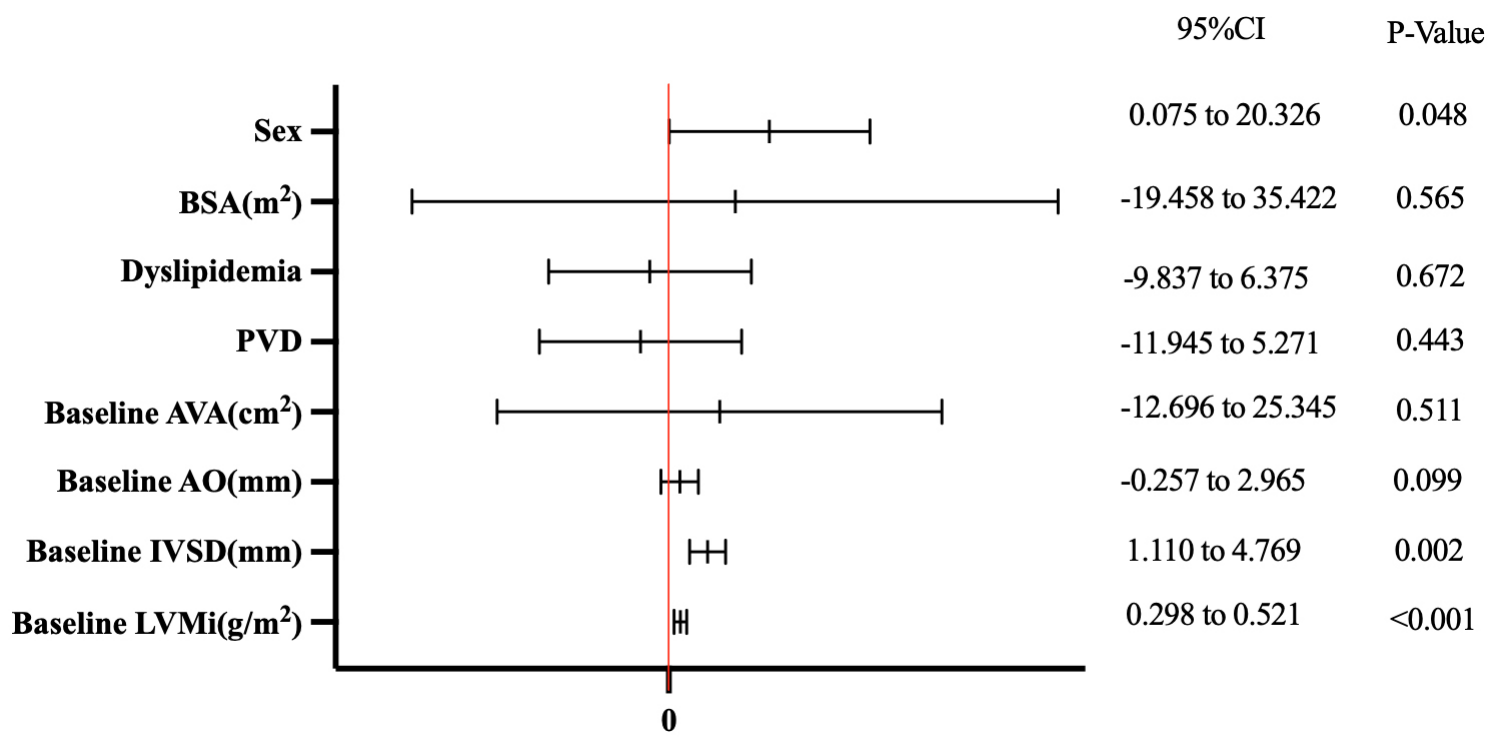
**Factors associated with mid-term regression of LVMi (ΔLVMi within 6 months) after TAVR**. BSA, body surface area; PVD, peripheral vascular disease; AVA, aortic valve area; AO, aortic root diameter; IVSD, interventricular septum in diastole; LVMi, left ventricular mass index; TAVR, transcatheter aortic valve replacement.

Table 4 presents the results of the multivariable linear-regression analysis, which assessed the independent predictors of greater mid-term LV mass regression post-TAVR. After adjusting for sex, BSA, dyslipidemia, PVD, AVA, AO, IVSD, and baseline LVMi, only sex, IVSD, and baseline LVMi were independently associated with greater mid-term LVMi regression post-TAVR. Multivariable linear-regression analysis showed only female patients (Beta: 10.200; 95% CI: 0.075–20.326; *p* = 0.048), higher IVSD (Beta: 2.939; 95% CI: 1.110–4.769; *p* = 0.002), and higher baseline LVMi (Beta: 0.409; 95% CI: 0.298–0.521; *p* < 0.001) were independently associated with greater mid-term LVMi regression post-TAVR, indicating that the female sex is an independent predictor for favorable mid-term LV remodeling after TAVR, as shown in Fig. 3. The studies comparing the cardiac structure and function after TAVR in male and female patients from different countries are shown in **Supplementary Table 1**.

**Table 4.** **Factors associated with early regression of the LVMi within 6 months**.

	Model 1			Model 2		
	Beta	95% CI	*p* value	Beta	95% CI	*p* value
Sex	–1.424	–12.388 to 9.539	0.797	10.200	0.075 to 20.326	**0.048**
BSA (m^2^)	–6.089	–36.496 to 24.317	0.692	7.982	–19.458 to 35.422	0.565
Dyslipidemia	–2.740	–13.879 to 8.399	0.627	–1.731	–9.837 to 6.375	0.672
PVD	5.402	–6.535 to 17.339	0.371	–3.337	–11.945 to 5.271	0.443
Baseline AVA (cm^2^)	–9.637	–35.015 to 15.741	0.453	6.324	–12.696 to 25.345	0.511
Baseline AO (mm)	2.431	0.325 to 4.537	**0.024**	1.354	–0.257 to 2.965	0.099
Baseline IVSD (mm)	5.670	3.716 to 7.624	< **0.001**	2.939	1.110 to 4.769	**0.002**
Baseline LVMi (g/m^2^)	0.474	0.381 to 0.568	< **0.001**	0.409	0.298 to 0.521	< **0.001**

Model 1. Crude analysis; Model 2. Adjusted for sex, BSA, dyslipidemia, PVD, baseline AVA, baseline AO, baseline IVSD, baseline LVMi; BSA, body surface area; PVD, peripheral vascular disease; AVA, aortic valve area; AO, aortic root diameter; IVSD, interventricular septum in diastole; LVMi, left ventricular mass index; *p* values in bold are statistically significant; F-test: F = 15.907, *p* < 0.001.

In conclusion, females with AS had favorable mid-term LV reverse remodeling post-TAVR compared to the males. The thicker IVSD and the more severe LV diastolic function before surgery, the greater the mid-term LV reverse remodeling after surgery, as shown in Fig. 3.

## 4. Discussion

Although the TAVR procedure has been increasingly performed across Asian countries, the use of the TAVR procedure is still common in the Chinese population. The present study provides several valuable insights into the procedure of TAVR and its interaction with sex. This consecutive cohort study evaluated the trends in LV remodeling in patients who underwent isolated TAVR according to sex. The major findings of this study are as follows: (1) At baseline, females had smaller BSA, AVA, LVEDD, IVSD, and lower rates of dyslipidemia and PVD compared to males; (2) Males exhibited larger aortic root structures and received larger prosthetic valves, although indexed AVA did not differ significantly between sexes; (3) Sex-specific differences in echocardiographic parameters (AVA, LVEDD, AO, IVSD) were observed throughout follow-up, with females showing greater improvements post-TAVR; (4) LVEF improved significantly only at 1-month follow-up in females; (5) Females demonstrated more favorable mid-term LV reverse remodeling (ΔLVMi within six months) than males; (6) Greater mid-term LV reverse remodeling (ΔLVMi) was associated with thicker preoperative IVSD and more severe LV diastolic dysfunction.

### 4.1 Clinical Presentation

Our study showed that women tend to have a lower incidence of dyslipidemia and PVD at baseline. Sex plays an important role in the development of AS, which leads to sex-specific differences with respect to the modulation of pathological processes [27, 28, 29]. Previous studies have demonstrated that almost half of females with severe AS are asymptomatic, resulting in a lower rate of diagnosis and treatment [30]. A study has shown that older females are associated with a higher incidence of symptomatic heart failure [27]. Consistent with previous findings [17, 31] that males tend to have a higher incidence of comorbidities, our study also found significant sex-specific differences in comorbidities, such as dyslipidemia and peripheral artery disease.

Women with smaller AO at baseline had a smaller size of valve implantation during TAVR were found in our study. A similar conclusion has been reached by a previous study on the very small BSA and aortic annulus in female Asians [32]. As shown in a previous study, the prostheses used for females tended to be smaller in size because of their smaller physical stature [33]. The sex-related differences in AO measurements were in accordance with recent computed tomography (CT) studies, which demonstrated larger diameters in male patients. The diameters were measured across multiplanar planes from the supra-annular to the subannular levels [34, 35]. This should be the focus of interobserver analysis because subjective measurements of AO are needed [36]. Thus, preprocedural measurements of aorta ascendens should be carefully standardized and further refined to understand the influence of sex. A larger type of aortic root structures in male patients might contribute to a higher incidence of valve-in-valve implantation after TAVR. Valve-in-valve implantation has already been known to be a higher technical complication. A previous study has demonstrated that choosing a prosthetic valve with moderate oversizing and more thorough measurement of anatomy for male patients may lead to better surgical outcomes [37].

### 4.2 Pathophysiology and Anatomy

In our cohort, women had favorable mid-term LV reverse remodeling (ΔLVMi within six months) post-TAVR compared to males. Sex-specific outcomes, especially AVA, LVEDD, AO, IVSD were significantly different during each follow-up within six-month, which indicated that females had greater improvements after TAVR in those echocardiographic characteristics. LVEF only improved significantly at 1-month follow-up in women compared to men. Multiple studies have shown that different effects of chronic afterload on the LV are associated with sex [10, 38]. Females who were diagnosed with severe AS appeared to have better characteristic of systolic function and slower progression of myocardial fibrosis than males did [14, 39]. Stangl *et al*. [40] concluded that while the regression of LV hypertrophy emerged in both males and females before TAVR, the improvement in LVEF was statistically significant only in women. Stangl *et al*. [40] confirmed that LV reverse remodeling occurs in both females and males 3 months post-TAVR, but the regression of LVEF was statistically significant only in female patients. Chen *et al*. [11] reported that early reverse regression of LVMi occurred in female patients. Lindman *et al*. [41] demonstrated that LVMi regression is statistically significant during the first-year follow-up post-TAVR in both male and female patients. The LVMi regressions of both groups exhibited similar incremental regressions and patterns. However, women were more likely to have LVMi regression (*p* = 0.004) [41]. Stangl *et al*. [40] showed that both LVM and LVMi regression significantly decreased during three-month follow-up post-TAVR, but there were no relevant differences between men and women. Ninomiya *et al*. [42] reported that incidence of LV reverse remodeling was significantly higher in men than in women. Kuneman *et al*. [43, 44] demonstrated that men and women with severe AS exhibited comparable improvements in LVEF, as well as similar reductions in LV volumes and LVMi at both 6-and 12-month following TAVR. The superior outcomes observed in women post-TAVR are not linked to sex-based disparities in LV reverse remodeling [43, 44, 45].

The obstruction resulting from aortic stenosis leads to pressure overload on the LV, prompting the development of concentric myocardial hypertrophy as an adaptive response to reduce wall stress [46, 47]. In the early stages of the disease, these alterations contribute to diastolic dysfunction by diminishing LV compliance, while systolic function remains relatively preserved. However, as the condition progresses, myocardial contractile function and deformation become compromised, ultimately leading to decreased cardiac output. These changes can be effectively monitored through accurate assessment of trans-valvular Doppler velocities and pressure gradients, both of which decrease as systolic function deteriorates significantly. Additionally, the left atrium undergoes morphological changes mirroring those of the LV, enlarging in response to chronic pressure overload [48]. This, in turn, results in elevated pulmonary venous and arterial pressures, culminating in heart failure.

Simard *et al*. [49] and Treibel *et al*. [50] reported that extracellular matrix expansion and myocardial fibrosis were detected in males with severe AS [33]. Cardiac magnetic resonance (CMR) and late gadolinium enhancement (LGE) could be used for identifying and quantifying cardiac muscle fibrosis to estimate LV remodeling [51, 52]. The superimposed pressure load on the LV caused by severe AS leads to hypertrophy in musculus cardiacus, resulting in structural changes in the LV. An acute decrease in the MPG caused by the TAVR may lead to LV unloading. The release of pressure overload in the LV may ultimately reverse LV remodeling and improve clinical outcomes [53, 54]. The study of sex-related mechanisms, including cellular, molecular and neurohormonal mechanisms, has been proposed. A previous study indicated not only increased interstitial fibrosis, increased proinflammatory pathway activity and increased profibrotic activation but also showed that there was differential expression of estrogen and androgen receptors [14, 16, 55, 56]. Other studies have shown that the increase in cardiac fibrosis observed in male patients with severe AS is related to increased SMAD family member 2 (SMAD2) phosphorylation and TGF-β1 protein expression [16, 57]. It is likely that sex-related differences may lead to differences in aortic stenosis pathology before TAVR, and further study of sex-related differences with respect to LV reverse remodeling after TAVR is needed. Although our findings suggest a potential sex-related difference in LV remodeling, the mechanisms underlying this observation—particularly in relation to myocardial fibrosis—remain speculative in the absence of direct histological or advanced imaging data. Future studies incorporating cardiac magnetic resonance imaging or myocardial biopsy could provide more definitive insights into the role of myocardial fibrosis in sex-specific remodeling patterns following TAVR.

The frequency of AS and transthyretin-related amyloid cardiomyopathy (ATTR-CM) increases with age. ATTR-CM can be found in 4 to 16% of the patients with aortic stenosis [58]. This condition profoundly affects the outcome which may lead to sex differences in AS pathology before TAVR and LV reverse remodeling after TAVR. This overlap is not coincidental. Both conditions share common demographic risk factors such as advanced age and male sex, and may present with similar clinical manifestations, including heart failure with preserved ejection fraction (HFpEF), increased LVWT, and low-flow, low-gradient AS [59]. ATTR-CM is an increasingly recognized form of infiltrative cardiomyopathy caused by the deposition of misfolded transthyretin (TTR) protein fibrils in the myocardial extracellular space. There are two main types: wild-type (ATTRwt), which primarily affects elderly individuals, and hereditary or variant (ATTRv), which is linked to TTR gene mutations [58]. ATTR-CM itself does not directly cause aortic valve stenosis, but it is often associated with it, particularly in elderly patients. The connection between the two conditions is believed to arise from shared age-related degenerative processes. In wild-type transthyretin amyloidosis (ATTRwt), misfolded transthyretin proteins are deposited not only in the myocardium but also in valvular tissue, including the aortic valve. This can contribute to valvular thickening, fibrosis, and calcification, which are key pathological features of aortic stenosis. Additionally, chronic pressure overload from AS may accelerate myocardial stress and promote amyloid deposition in the heart, creating a vicious cycle. Thus, in aging individuals—especially men—it is common to find both AS and ATTR-CM coexisting due to overlapping mechanisms involving senile systemic amyloidosis, degenerative valve disease, and age-related cardiac remodeling [60]. The presence of ATTR-CM in patients with AS especially males is clinically significant because it can influence both treatment strategies and prognosis. Patients with dual pathology (AS + ATTR-CM + males) tend to have worse outcomes after valve replacement compared to those with isolated AS, including higher rates of persistent heart failure symptoms, reduced functional recovery, and increased mortality. In the context of the Chinese population, data are limited, but the aging demographics suggest that ATTR-CM may also be underdiagnosed among elderly Chinese patients with AS. As such, further studies are needed to explore its prevalence and clinical impact in this population, and to determine whether systematic screening could improve patient management and outcomes.

### 4.3 LVM Regression and LV Reverse Remodeling

In line with our imaging studies, the thicker IVSD and the more severe LV diastolic function before surgery, the greater the mid-term LV reverse remodeling after surgery in our observations. The definition of LV hypertrophy and LV diastolic dysfunction as increasing LVMi. LV pressure overload is caused by AS through a continuous increase in valvular resistance, resulting in structural changes in the LV. The superimposed pressure applied to the LV caused by AS may ultimately lead to structural changes in the LV, which indicates LV hypertrophy. The MPG offers the most value for assessing the degree of AS [61]. Patients with higher MPG at baseline may also represent those with chronic and severe cardiac muscle fibrosis. Patients with higher LVMi at baseline were more likely to have underlying myocardial fibrosis [62]. A higher baseline LVMi may indicate that patients have a poor contractile reserve and substantial chronic myocardial damage due to LV pressure overload. E.K. Sim *et al*. [63] concluded that the extent of LVM regression may differ among individuals. The extent to which LVM regresses is affected by sex, age, the prosthetic valve size, and the prosthesis-patient mismatch. Hypertrophy is usually associated with increased fibrosis and decreased structural reversibility due to long-term overload [64]. Myocardial fibrosis commonly occurs in response to myocyte apoptosis, replacement fibrosis and expansion of the extracellular space in most patients with severe AS [65]. The early phase of LVM tends to regress because of the relief of LV pressure overload. The early phase of regression of myocardial edema only lasts several months, but it takes years for the late phase of LVM regression because of remodeling of interstitial fibrosis.

Although our study found the outcomes of TAVR procedures are different between different gender, this study has some limitations. First, our research involved a single-center study and a small-sized registry study. All clinical event data were obtained via review of medical records and telephone interviews. However, Firth’s correction was used for revision in our study due to its small sample size. Therefore, we believe that our use of multivariable analysis is statistically justifiable and provides valid insights within the limitations of our cohort. A further study with multicenter, large-scale, long-term follow-up cohorts and clinical outcomes would be better for evaluating LVMi regression. Second, the patients in our study did not undergo routine CMR. CMR offers a more accurate measurement of LVH and cardiac muscle fibrosis than echocardiography. The CMR is the gold standard for evaluating LV remodeling. However, further studies via CMR data are needed to determine the relationships between myocardial fibrosis and changes in LVMi. Third, further studies concerning the complications of TAVR are needed to assess the sex-related differences in reverse LV remodeling. In addition, based on echocardiographic measurements of LVM and RWT, four patterns were defined: normal geometry (NG), concentric remodeling (CR), concentric hypertrophy (CH), and eccentric hypertrophy (EH). Yet, the effect of different LV geometry was not determined. Due to the small sample size, it is challenging to perform statistical analysis based on the four cardiac remodeling patterns (NG, CR, CH, EH). In future studies, further studies enrolling a large number of patients with different LV geometry of patients who underwent TAVR are warranted to facilitate a more comprehensive classification and analysis. Moreover, although data specific to the Chinese population are limited, the aging trend suggests that ATTR-CM may be under-recognized in elderly Chinese patients with aortic stenosis. Therefore, additional research is warranted to investigate its prevalence, clinical significance, and the potential influence of sex-related differences on management strategies and patient outcomes.

## 5. Conclusions

This consecutive cohort study evaluated the trends in LV remodeling in patients who underwent isolated TAVR according to sex in a Chinese Population. The following major findings were identified: (1) females had a smaller BSA, AVA, LVEDD, IVSD and a lower incidence of dyslipidemia and PVD at baseline; (2) men had a larger aortic root structures at baseline and a larger size of valve implantation during the procedure, although indexed AVA was not significantly different between the two groups; (3) sex-specific outcomes, especially AVA, LVEDD, AO, IVSD were significantly different during each follow-up, which indicated that females had greater improvements after TAVR in those echocardiographic characteristics; (4) LVEF only improved significantly at 1-month follow-up in females compared to males; (5) women had favorable mid-term LV reverse remodeling (ΔLVMi within six months) post-TAVR compared to men; and (6) the thicker IVSD and the more severe LV diastolic function before surgery, the greater the mid-term LV reverse remodeling (ΔLVMi within six months) after surgery.

TAVR in both groups appeared to be safe, and feasible according to our study in a Chinese Population. Multivariable linear-regression analysis showed only that female patients, higher IVSD, and higher baseline LVMi were independently associated with greater mid-term LVMi regression post-TAVR, indicating that the female sex is an independent predictor for favorable mid-term LV remodeling after TAVR.

## Availability of Data and Materials

The data sets analyzed during the current study are not publicly available due to restrictions apply to the availability of these data but are available from the corresponding authors on reasonable request.

## Supplementary Material

Supplementary material associated with this article can be found, in the online version, at https://doi.org/10.31083/RCM39581.

## References

[1] Smith CR, Leon MB, Mack MJ, Miller DC, Moses JW, Svensson LG (2011). Transcatheter versus surgical aortic-valve replacement in high-risk patients. *The New England Journal of Medicine*.

[2] Leon MB, Smith CR, Mack MJ, Makkar RR, Svensson LG, Kodali SK (2016). Transcatheter or Surgical Aortic-Valve Replacement in Intermediate-Risk Patients. *The New England Journal of Medicine*.

[3] Mack MJ, Leon MB, Thourani VH, Makkar R, Kodali SK, Russo M (2019). Transcatheter Aortic-Valve Replacement with a Balloon-Expandable Valve in Low-Risk Patients. *The New England Journal of Medicine*.

[4] Popma JJ, Deeb GM, Yakubov SJ, Mumtaz M, Gada H, O’Hair D (2019). Transcatheter Aortic-Valve Replacement with a Self-Expanding Valve in Low-Risk Patients. *The New England Journal of Medicine*.

[5] Baumgartner H, Falk V, Bax JJ, De Bonis M, Hamm C, Holm PJ (2017). 2017 ESC/EACTS Guidelines for the management of valvular heart disease. *European Heart Journal*.

[6] Amgai B, Chakraborty S, Bandyopadhyay D, Gupta M, Patel N, Hajra A (2021). Sex Differences in In-Hospital Outcomes of Transcatheter Aortic Valve Replacement. *Current Problems in Cardiology*.

[7] Manoharan G, Van Mieghem NM, Windecker S, Bosmans J, Bleiziffer S, Modine T (2018). 1-Year Outcomes With the Evolut R Self-Expanding Transcatheter Aortic Valve: From the International FORWARD Study. *JACC. Cardiovascular Interventions*.

[8] Singh A, Musa TA, Treibel TA, Vassiliou VS, Captur G, Chin C (2019). Sex differences in left ventricular remodelling, myocardial fibrosis and mortality after aortic valve replacement. *Heart*.

[9] O’Connor SA, Morice MC, Gilard M, Leon MB, Webb JG, Dvir D (2015). Revisiting Sex Equality With Transcatheter Aortic Valve Replacement Outcomes: A Collaborative, Patient-Level Meta-Analysis of 11,310 Patients. *Journal of the American College of Cardiology*.

[10] Piro M, Della Bona R, Abbate A, Biasucci LM, Crea F (2010). Sex-related differences in myocardial remodeling. *Journal of the American College of Cardiology*.

[11] Chen SC, Leu HB, Chang HH, Chen IM, Chen PL, Lin SM (2020). Women had favourable reverse left ventricle remodelling after TAVR. *European Journal of Clinical Investigation*.

[12] Walczewski M, Gasecka A, Huczek Z, Rymuza B, Kochman J (2021). Ten-year experience with transcatheter aortic valve implantation in bicuspid aortic valve: lessons learned and future perspectives. *Postepy W Kardiologii Interwencyjnej = Advances in Interventional Cardiology*.

[13] Hayashida K, Morice MC, Chevalier B, Hovasse T, Romano M, Garot P (2012). Sex-related differences in clinical presentation and outcome of transcatheter aortic valve implantation for severe aortic stenosis. *Journal of the American College of Cardiology*.

[14] Kararigas G, Dworatzek E, Petrov G, Summer H, Schulze TM, Baczko I (2014). Sex-dependent regulation of fibrosis and inflammation in human left ventricular remodelling under pressure overload. *European Journal of Heart Failure*.

[15] Aurigemma GP, Gaasch WH (1995). Gender differences in older patients with pressure-overload hypertrophy of the left ventricle. *Cardiology*.

[16] Petrov G, Regitz-Zagrosek V, Lehmkuhl E, Krabatsch T, Dunkel A, Dandel M (2010). Regression of myocardial hypertrophy after aortic valve replacement: faster in women?. *Circulation*.

[17] Chandrasekhar J, Dangas G, Mehran R (2017). Valvular Heart Disease in Women, Differential Remodeling, and Response to New Therapies. *Current Treatment Options in Cardiovascular Medicine*.

[18] van Bergeijk KH, van Ginkel DJ, Brouwer J, Nijenhuis VJ, van der Werf HW, van den Heuvel AFM (2023). Sex Differences in Outcomes After Transcatheter Aortic Valve Replacement: A POPular TAVI Subanalysis. *JACC. Cardiovascular Interventions*.

[19] Strange JE, Holt A, Christensen DM, Nouhravesh N, Petersen JK, Bække PS (2024). End of Life After Transcatheter Aortic Valve Replacement: A Danish Nationwide Cohort Study. *JACC. Cardiovascular Interventions*.

[20] Nakase M, Tomii D, Maznyczka A, Samim D, Lanz J, Praz F (2024). Sex-Specific Differences in Upstream Cardiac Damage in Patients With Aortic Stenosis Undergoing TAVR. *JACC. Cardiovascular Interventions*.

[21] Prosperi-Porta G, Nguyen V, Willner N, Dreyfus J, Eltchaninoff H, Burwash IG (2023). Association of Age and Sex With Use of Transcatheter Aortic Valve Replacement in France. *Journal of the American College of Cardiology*.

[22] Jilaihawi H, Wu Y, Yang Y, Xu L, Chen M, Wang J (2015). Morphological characteristics of severe aortic stenosis in China: imaging corelab observations from the first Chinese transcatheter aortic valve trial. *Catheterization and Cardiovascular Interventions: Official Journal of the Society for Cardiac Angiography & Interventions*.

[23] Shishido K, Yamanaka F, Ochiai T, Moriyama N, Yokoyama H, Yokota S (2021). Effect of Sex on Mortality and Left Ventricular Remodeling After Transcatheter Aortic Valve Implantation. *Circulation Journal: Official Journal of the Japanese Circulation Society*.

[24] Lang RM, Bierig M, Devereux RB, Flachskampf FA, Foster E, Pellikka PA (2005). Recommendations for chamber quantification: a report from the American Society of Echocardiography’s Guidelines and Standards Committee and the Chamber Quantification Writing Group, developed in conjunction with the European Association of Echocardiography, a branch of the European Society of Cardiology. *Journal of the American Society of Echocardiography: Official Publication of the American Society of Echocardiography*.

[25] Lang RM, Badano LP, Mor-Avi V, Afilalo J, Armstrong A, Ernande L (2015). Recommendations for cardiac chamber quantification by echocardiography in adults: an update from the American Society of Echocardiography and the European Association of Cardiovascular Imaging. *Journal of the American Society of Echocardiography: Official Publication of the American Society of Echocardiography*.

[26] Kuneman JH, Singh GK, Hansson NC, Fusini L, Poulsen SH, Fortuni F (2022). Subclinical leaflet thrombosis after transcatheter aortic valve implantation: no association with left ventricular reverse remodeling at 1-year follow-up. *The International Journal of Cardiovascular Imaging*.

[27] Sevilla T, Ramos N, Carnero M, Amat-Santos IJ, Carrasco-Moraleja M, Revilla A (2023). Sex Differences in Clinical Outcomes after Aortic Valve Intervention for Isolated Severe Aortic Stenosis. *Journal of Clinical Medicine*.

[28] Summerhill VI, Moschetta D, Orekhov AN, Poggio P, Myasoedova VA (2020). Sex-Specific Features of Calcific Aortic Valve Disease. *International Journal of Molecular Sciences*.

[29] Tribouilloy C, Bohbot Y, Rusinaru D, Belkhir K, Diouf M, Altes A (2021). Excess Mortality and Undertreatment of Women With Severe Aortic Stenosis. *Journal of the American Heart Association*.

[30] Généreux P, Stone GW, O’Gara PT, Marquis-Gravel G, Redfors B, Giustino G (2016). Natural History, Diagnostic Approaches, and Therapeutic Strategies for Patients With Asymptomatic Severe Aortic Stenosis. *Journal of the American College of Cardiology*.

[31] Steeds RP, Messika-Zeitoun D, Thambyrajah J, Serra A, Schulz E, Maly J (2021). IMPULSE: the impact of gender on the presentation and management of aortic stenosis across Europe. *Open Heart*.

[32] Chiam PTL, Hayashida K, Watanabe Y, Yin WH, Kao HL, Lee MKY (2021). Sex differences in patients undergoing transcatheter aortic valve replacement in Asia. *Open Heart*.

[33] Chiam PTL, Koh AS, Ewe SH, Sin YK, Chao VTT, Ng CK (2013). Iliofemoral anatomy among Asians: implications for transcatheter aortic valve implantation. *International Journal of Cardiology*.

[34] Aggarwal SR, Clavel MA, Messika-Zeitoun D, Cueff C, Malouf J, Araoz PA (2013). Sex differences in aortic valve calcification measured by multidetector computed tomography in aortic stenosis. *Circulation. Cardiovascular Imaging*.

[35] Hamdan A, Barbash I, Schwammenthal E, Segev A, Kornowski R, Assali A (2017). Sex differences in aortic root and vascular anatomy in patients undergoing transcatheter aortic valve implantation: A computed-tomographic study. *Journal of Cardiovascular Computed Tomography*.

[36] He JJ, Xiong TY, Yao YJ, Peng Y, Wei JF, He S (2022). Sex Difference in Outcomes Following Transcatheter Aortic Valve Replacement in Bicuspid Aortic Stenosis. *JACC. Cardiovascular Interventions*.

[37] Walther T, Hamm CW, Schuler G, Berkowitsch A, Kötting J, Mangner N (2015). Perioperative Results and Complications in 15,964 Transcatheter Aortic Valve Replacements: Prospective Data From the GARY Registry. *Journal of the American College of Cardiology*.

[38] Scantlebury DC, Borlaug BA (2011). Why are women more likely than men to develop heart failure with preserved ejection fraction?. *Current Opinion in Cardiology*.

[39] Carroll JD, Carroll EP, Feldman T, Ward DM, Lang RM, McGaughey D (1992). Sex-associated differences in left ventricular function in aortic stenosis of the elderly. *Circulation*.

[40] Stangl V, Baldenhofer G, Knebel F, Zhang K, Sanad W, Spethmann S (2012). Impact of gender on three-month outcome and left ventricular remodeling after transfemoral transcatheter aortic valve implantation. *The American Journal of Cardiology*.

[41] Lindman BR, Stewart WJ, Pibarot P, Hahn RT, Otto CM, Xu K (2014). Early regression of severe left ventricular hypertrophy after transcatheter aortic valve replacement is associated with decreased hospitalizations. *JACC. Cardiovascular Interventions*.

[42] Ninomiya R, Orii M, Fujiwara J, Yoshizawa M, Nakajima Y, Ishikawa Y (2020). Sex-Related Differences in Cardiac Remodeling and Reverse Remodeling After Transcatheter Aortic Valve Implantation in Patients with Severe Aortic Stenosis in a Japanese Population. *International Heart Journal*.

[43] Kuneman JH, Singh GK, Milhorini Pio S, Hirasawa K, Hautemann D, van der Kley F (2022). Sex differences in left ventricular remodelling in patients with severe aortic valve stenosis. *European Heart Journal. Cardiovascular Imaging*.

[44] Kuneman JH, Butcher SC, Stassen J, Singh GK, Pio SM, van der Kley F (2022). Interaction between sex and left ventricular reverse remodeling and its association with outcomes after transcatheter aortic valve implantation. *The International Journal of Cardiovascular Imaging*.

[45] Itzhaki Ben Zadok O, Kornowski R, Finkelstein A, Barbash I, Danenberg H, Segev A (2019). Temporal Trends in Gender-Related Differences and Outcomes in Patients Who Underwent Transcatheter Aortic Valve Implantation (from the Israeli Transcatheter Aortic Valve Implantation Multicenter Registry). *The American Journal of Cardiology*.

[46] Lisi M, Pastore MC, Fiorio A, Cameli M, Mandoli GE, Righini FM (2022). Left Atrial Remodeling in Response to Aortic Valve Replacement: Pathophysiology and Myocardial Strain Analysis. *Life (Basel, Switzerland)*.

[47] Baumgartner H, Hung J, Bermejo J, Chambers JB, Evangelista A, Griffin BP (2009). Echocardiographic assessment of valve stenosis: EAE/ASE recommendations for clinical practice. *Journal of the American Society of Echocardiography: Official Publication of the American Society of Echocardiography*.

[48] Henein MY, Holmgren A, Lindqvist P (2015). Left atrial function in volume versus pressure overloaded left atrium. *The International Journal of Cardiovascular Imaging*.

[49] Simard L, Côté N, Dagenais F, Mathieu P, Couture C, Trahan S (2017). Sex-Related Discordance Between Aortic Valve Calcification and Hemodynamic Severity of Aortic Stenosis: Is Valvular Fibrosis the Explanation?. *Circulation Research*.

[50] Treibel TA, Kozor R, Fontana M, Torlasco C, Reant P, Badiani S (2018). Sex Dimorphism in the Myocardial Response to Aortic Stenosis. *JACC. Cardiovascular Imaging*.

[51] Dweck MR, Joshi S, Murigu T, Alpendurada F, Jabbour A, Melina G (2011). Midwall fibrosis is an independent predictor of mortality in patients with aortic stenosis. *Journal of the American College of Cardiology*.

[52] Barone-Rochette G, Piérard S, De Meester de Ravenstein C, Seldrum S, Melchior J, Maes F (2014). Prognostic significance of LGE by CMR in aortic stenosis patients undergoing valve replacement. *Journal of the American College of Cardiology*.

[53] Une D, Mesana L, Chan V, Maklin M, Chan R, Masters RG (2015). Clinical Impact of Changes in Left Ventricular Function After Aortic Valve Replacement: Analysis From 3112 Patients. *Circulation*.

[54] Levy D, Garrison RJ, Savage DD, Kannel WB, Castelli WP (1990). Prognostic implications of echocardiographically determined left ventricular mass in the Framingham Heart Study. *The New England Journal of Medicine*.

[55] Lau ES, Kaur G, Sharma G (2023). Sex and Age Differences in Myocardial Fibrosis: Do Sex Hormones Address the Knowledge Gap?. *JACC. Advances*.

[56] Marsh JD, Lehmann MH, Ritchie RH, Gwathmey JK, Green GE, Schiebinger RJ (1998). Androgen receptors mediate hypertrophy in cardiac myocytes. *Circulation*.

[57] Villar AV, Llano M, Cobo M, Expósito V, Merino R, Martín-Durán R (2009). Gender differences of echocardiographic and gene expression patterns in human pressure overload left ventricular hypertrophy. *Journal of Molecular and Cellular Cardiology*.

[58] Antonopoulos AS, Panagiotopoulos I, Kouroutzoglou A, Koutsis G, Toskas P, Lazaros G (2022). Prevalence and clinical outcomes of transthyretin amyloidosis: a systematic review and meta-analysis. *European Journal of Heart Failure*.

[59] Cheng R, Griffin J (2022). Implications of screening for coexisting transthyretin amyloidosis and aortic stenosis. *Heart (British Cardiac Society)*.

[60] Takashio S, Yamada T, Nishi M, Morioka M, Fujiyama A, Nakashima N (2022). Sex-related differences in the clinical characteristics of wild-type transthyretin amyloidosis cardiomyopathy. *Journal of Cardiology*.

[61] Corrigan FE, Zhou X, Lisko JC, Hayek SS, Parastatidis I, Keegan P (2018). Mean Aortic pressure gradient and global longitudinal strain recovery after transcatheter aortic valve replacement - A retrospective analysis. *Hellenic Journal of Cardiology: HJC = Hellenike Kardiologike Epitheorese*.

[62] Carluccio E, Biagioli P, Zuchi C, Bardelli G, Murrone A, Lauciello R (2016). Fibrosis assessment by integrated backscatter and its relationship with longitudinal deformation and diastolic function in heart failure with preserved ejection fraction. *The International Journal of Cardiovascular Imaging*.

[63] Sim EK, Orszulak TA, Schaff HV, Shub C (1994). Influence of prosthesis size on change in left ventricular mass following aortic valve replacement. *European Journal of Cardio-thoracic Surgery: Official Journal of the European Association for Cardio-thoracic Surgery*.

[64] Oh JK, Lee SH, Lee SA, Kang DY, Lee S, Kim HJ (2021). Prognostic impact of left ventricular mass regression after transcatheter aortic valve replacement in patients with left ventricular hypertrophy. *International Journal of Cardiology*.

[65] Hein S, Arnon E, Kostin S, Schönburg M, Elsässer A, Polyakova V (2003). Progression from compensated hypertrophy to failure in the pressure-overloaded human heart: structural deterioration and compensatory mechanisms. *Circulation*.

