# Supplementary material for: Sex Differences in Reverse Left Ventricular Remodeling in Patients Who Underwent Transcatheter Aortic Valve Replacement in a Chinese Population
Source: Rev Cardiovasc Med. 2025 Aug 28;26(8):39581. doi: 10.31083/RCM39581 (PMC12415741; doi:10.31083/RCM39581)
Supplement: Supplementary file 1 [file 2153-8174-26-8-39581-s1.docx]

**Supplementary Table 1.** Studies comparing the cardiac structure and function after TAVR in male and female patients in different countries

| First author (year of publication) | Country | Follow-up period | Numbers of subjects | Main findings | Reference number |
| --- | --- | --- | --- | --- | --- |
| Stangl  et al. (2021) | Germany | 3 Months | 100 | After TAVR, regression of hypertrophy occurred in men and women, but improvement of the ejection fraction was significant only in women. | 40 |
| Lindman  et al. (2014) | America | 30 Days | 690 | Female sex was  independently associated with greater early LVMi regression. | 41 |
| Chen  et al. (2020) | China | 3 Months | 100 | Female sex is an  independent predictor for favourable LV remodelling after TAVR. | 11 |
| Ninomiya  et al. (2020) | Japan | 3 Months | 100 | Incidence of LV reverse remodeling was significantly higher in men than in women. | 42 |
| Kuneman  et al. (2021) | The Netherlands | 5.1 Years | 289 | No significant interaction was observed between outcome and the LV remodeling patterns and sex. | 43 |
| Kuneman  et al. (2022) | The Netherlands | 12 Months | 459 | Women showed better survival after TAVR as compared to men. The superior outcomes noted in women after TAVR are not associated with sex differences in LV reverse remodeling. | 44 |

TAVR, transcatheter aortic valve replacement; LVMi, left ventricular mass index; LV, left ventricle.
